# Angioleiomyoma, a rare intracranial tumor: 3 case report and a literature review

**DOI:** 10.1186/1477-7819-12-216

**Published:** 2014-07-16

**Authors:** Lijun Sun, Yan Zhu, Hong Wang

**Affiliations:** 1 Department of Neurosurgery, Tianjin Cerebral Vascular and Neural Degenerative Diseases Key Laboratory, Tianjin Huanhu Hospital, 122 Qixiangtai Street, 300060 Tianjin, China

**Keywords:** Angioleiomyoma, Cavernous sinus, Intracranial tumor, Magnetic resonance imaging, Vascular leiomyoma

## Abstract

Three cases of intracranial angioleiomyoma (ALM) in our neurosurgery center are reported in detail. ALM is a benign soft tissue tumor comprised of mature smooth muscle cells and a prominent vascular component, which is extremely rare as a primary intracranial lesion. Altogether, only 12 cases were recorded in the literature to date, to the best of our knowledge. Case 1 is the second report of intra-sella ALM, a 51-year-old woman presented with visual deterioration for 2 months. An MRI showed an intra-sella 3-cm tumor, partially flame-like, enhanced with gadolinium. Using microscopic endonasal transsphenoidal approach, the tumor was completely resected with great difficulty. At 11 days post-surgery, she died of a sudden catastrophic nasal hemorrhage. An angiography revealed a pseudo-aneurysm of ICA (internal carotid artery). Case 2 is a 49-year-old man who presented with weakness of the lower limbs for 1 year. A large subtentorial mass was found affixed to the torcular and straight sinus, which was partially flame-like, dramatically enhanced as in case 1. Case 3 is that of a 77-year-old man. An ALM mass was revealed in the diploe of left temporal bone, and had eroded the inner table. Immunohistochemical workup confirmed the diagnosis of angioleiomyoma in all 3 cases. The radiology, operation, and complication of surgery in each case were discussed. In conclusion, intracranial ALMs are extremely rare, usually located ex-neuro axis (such as in our cases), in the sella, in posterior fossa, or in the skull. Magnetic resonance imaging (MRI) revealed a special feature of flame-like partial enhancement that may be helpful to distinguish ALM from pituitary tumors or meningiomas, and should result in the consideration of this rare tumor entity early on in the diagnostic process. A definitive diagnosis depends on histological analyses. The resection of ALM in certain locations is difficult and risky because of the rich blood supply.

## Background

Angioleiomyomas (ALMs) are benign soft tissue tumors commonly occurring in middle-aged women as nodules in the subcutaneous tissue of the lower extremities [1], which is composed of mature smooth muscle cells and a prominent vascular component. But as intracranial lesions are extremely rare, only 12 cases were recorded in the literature to date, to the best of our knowledge. Most of the intracranial cases were in dura or at least in ex-neuro axis sites, such as the cavernous sinus dura [2,3], except 2 cases which were in other parenchymal locations such as the basal ganglia [4,5]. The lack of experience makes the preoperative diagnosis very difficult. Due to its benign nature, surgical excision is curative.

Our center admitted about 4403 cases of primary intracranial tumor in the last 5 years, only 3 of which (less than 0.1%) were identified as ALM. Case 1 is the second report of intra-sella ALM (the first was reported in 2010) [6], case 2 is a large subtentorial tumor, and case 3 is the first report of ALM in the skull vault. The magnetic resonance imaging (MRI) features were unusual and have potential to be helpful in distinguishing ALMs from pituitary tumors, meningiomas, and other tumors. A histopathology study revealed this characteristic figure. Although ALMs are very rarely encountered, it should be considered earlier in the diagnostic process, and be properly treated with caution.

## Case presentation

### Case 1

#### History and examination

**Figure 1:**
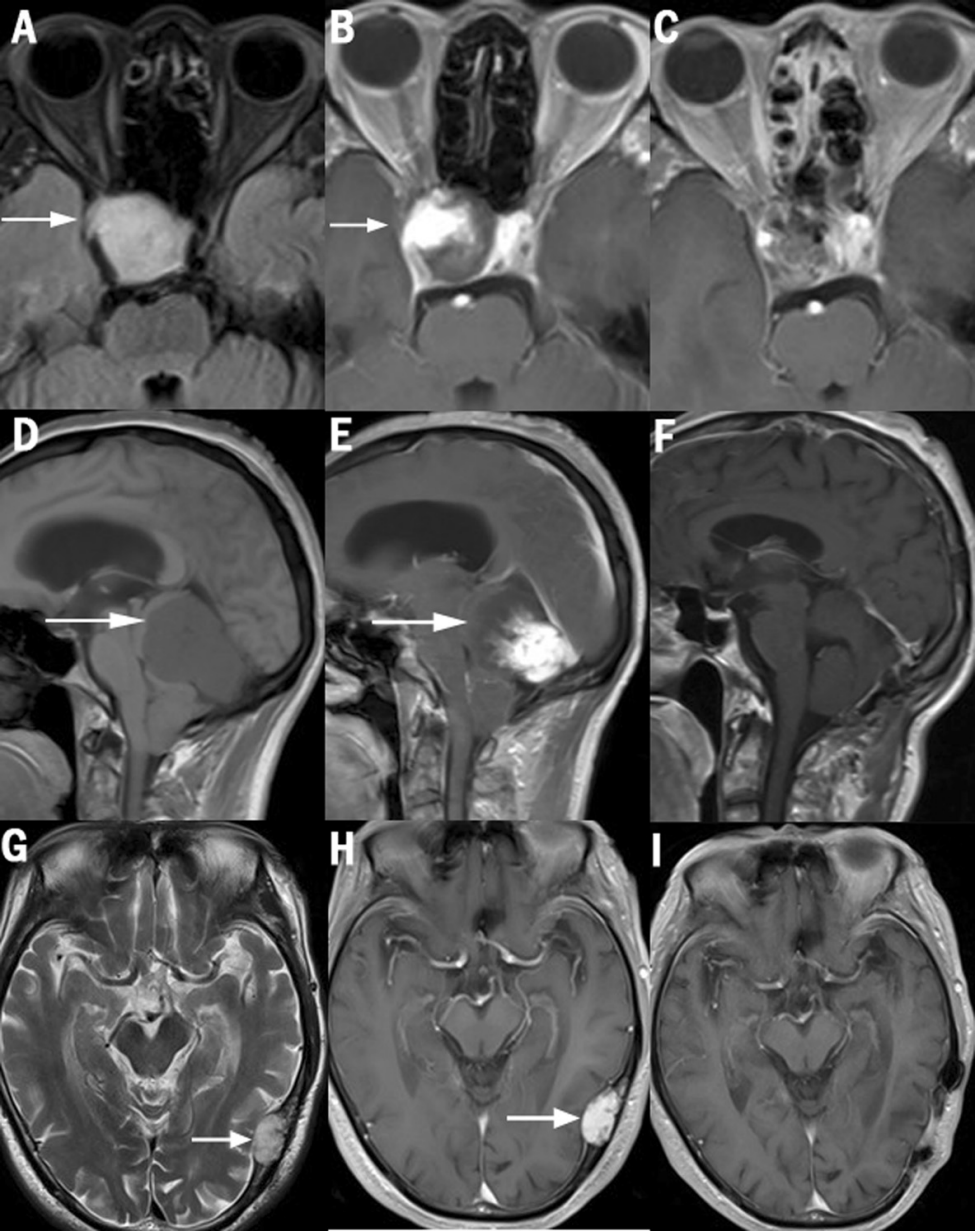
**MRI scans for cases 1-3.** Case 1: **(A)** preoperative MRI T2-w FLAIR, **(B)** T1-w MRI with contrast revealed a partially enhanced lesion in the sella, adjacent to the cavernous sinus, **(C)** postoperative MRI with contrast. Case 2: **(D)** preoperative MRI T1-w revealed a large subtentorial lesion, the cerebellum and brainstem were obviously compressed forward and downward, **(E)** MRI with contrast revealed the lesion was partially flame-like enhanced, **(F)** postoperative MRI with contrast one year later. Case 3: **(G)** preoperative T2-w MRI revealed a lesion in diploic space of the left temporal skull vault which had invaded inward, **(H)** MRI with contrast showed the tumor was enhanced, **(I)** postoperative MRI with contrast one year later. The arrows indicate the tumors. MRI (Magnetic resonance imaging), FLAIR (fluid-attenuated inversion recovery).

A 51-year-old woman presented to our neurosurgery center with a two-month long blurry of the right eye, which gradually worsened, eventually losing vision in the eye 20 days before admission. A neurological examination revealed that the sight of the right eye was 0.03, with bilateral temporal hemianopsia. There were no other remarkable signs. An endocrinological evaluation showed only a low value of blood cortisol. T1-weighted (T1-w) MRI scans revealed an iso-signal intra-sella tumor sized 3.0 × 2.5 × 2.5 cm, close to the medial part of the right cavernous sinus, with distinctive partial enhancement after a gadolinium injection, which arose from the right cavernous sinus, flame-like in shape, with bush-like edge (Figure 1B). T2-fluid-attenuated inversion recovery-weighted sequences (T2-FLAIR) showed a homogeneous hyper-signal tumor (Figure 1A). The sella fossa was obviously enlarged and the tumor had invaded into the sphenoid sinus. It was considered to be a non-functional pituitary tumor.

#### Operation and postoperative course

**Figure 2:**
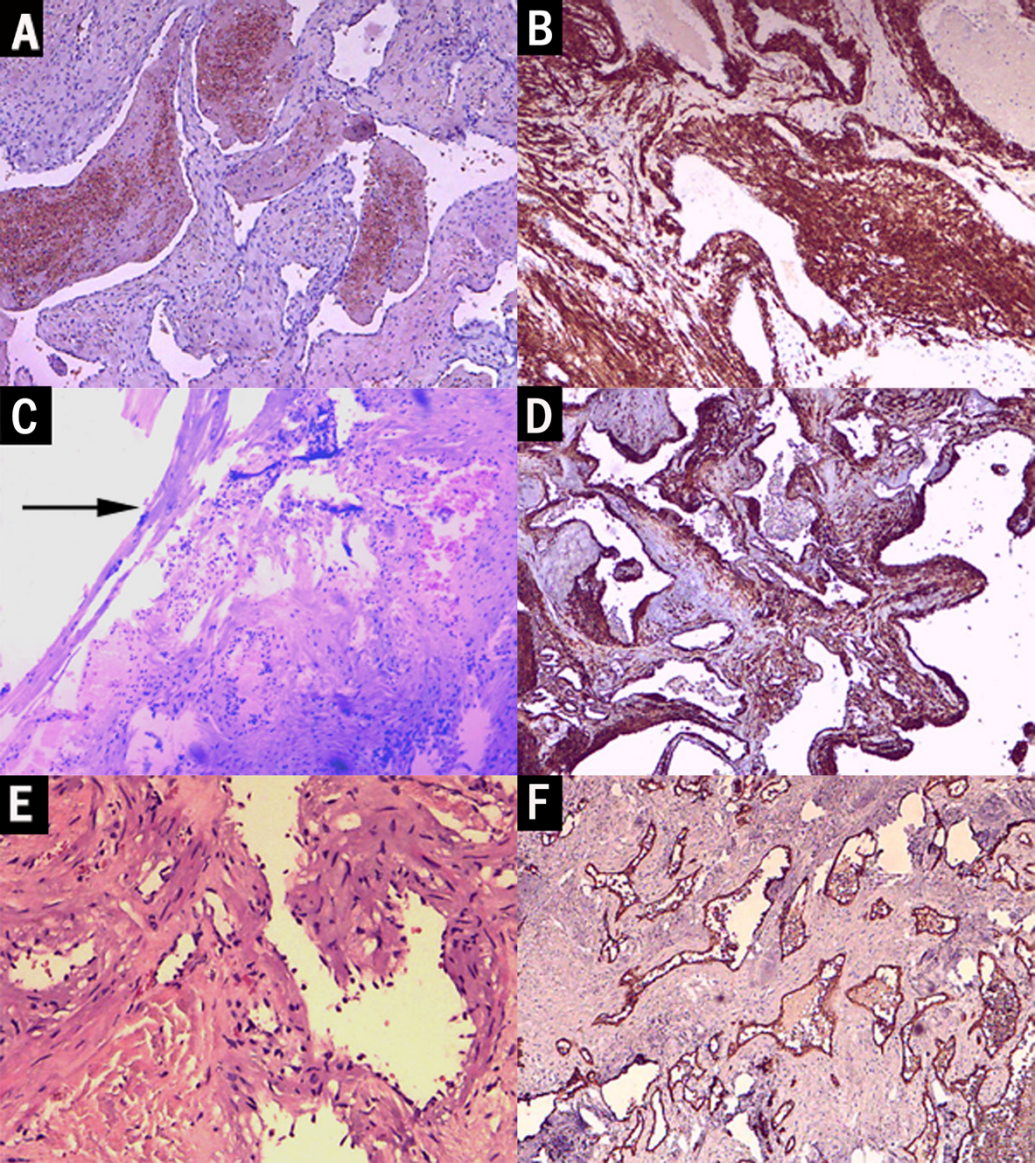
**Photomicrographs of the pathological specimen.** Case 1: **(A)** 40 × magnification photomicrograph delineating the overall view, hematoxylin and eosin (H&E). Loose arraying spindle cells riddled with irregular vascular spaces, some containing blood. **(B)** 40 × magnification immunohistochemistry of smooth muscle actin (SMA), the spindle cells were strongly positive for SMA as shown by the brown color. Case 2: **(C)** 40 × magnification photomicrograph, H&E, showed the interface between the tumor and dura. **(D)** 40 × magnification with immunostaining for SMA, whorls of spindle cells were positive for SMA, showing brown. Case 3: **(E)** 100 × magnification photomicrograph (H&E) illustrated that the tumor was composed of smooth muscle cells and vessels. **(F)** 40 × magnification photomicrograph of the immunohistochemistry of CD34 showed a selected stain of the single layer of endothelium lining the channels, shown by the brown color. The arrow points to the adjacent dura matter.

**Figure 3:**
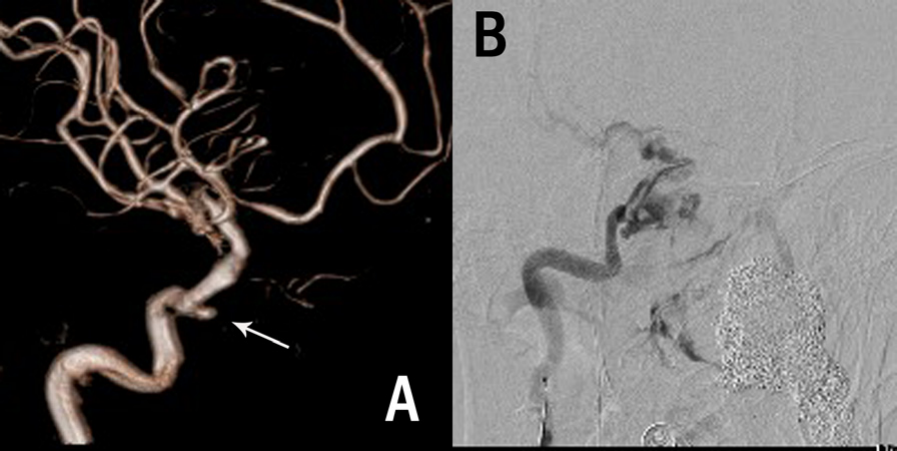
**The digital subtractive angiography (DSA) of Case 1 after a lethal nasal hemorrhage. (A)** The 3-dimension reconstruction DSA showed the pseudo-aneurysm (the arrow) at the cavernous segment of the right internal carotid artery (ICA), **(B)** the DSA roadmap when the second bleeding occurred.

A microscopic endonasal transsphenoidal approach was used for surgical resection. After dural opening, a solid, tenacious, purple mass was found, sticking firmly with surrounding structures, with abundant blood supply. The tumor was extremely difficult to manipulate and had to be excised *en bloc* to avoid bleeding. It was completely excised after hours of careful separation between the tumor capsule and the cavernous sinus. The removed tissue was dark purple, shrinking, sponge-like, and approximately 2.5 cm in size. After common hemostasis, the wound was sealed with gel foam and glue. Microscopically, the lesion was essentially composed of spindle shaped smooth muscle cells and blood vessels with thick walls devoid of elastic lamina (Figure 2A). The vessel lumen was slit-like and the inner wall was lined with monolayer endothelial cells, and the smooth muscles of the vascular wall intermingled with the smooth muscle cells between the vessels. No mitoses were found. Immunohistochemistry revealed that the spindle cells were positive for vimentin (Vim) and smooth muscle actin (SMA) (Figure 2B), and negative for glial fibrillary acidic protein (GFAP) and neurofilament (NF). The endothelial cells were positive for CD34. The tumor was identified as ALM. The patient had no complaints postoperatively, except for a slight headache. The sight of her right eye promoted obviously two days after surgery. An MRI scan demonstrated a total resection (Figure 1C) without any bleeding. At 11 days after the operation, she complained of a decreasing of hearing in her left ear and a little nose discharge, although the headache diminished. An ear-nose-throat inspection was recommended. However, an emergent incident happened while she was under nasal endoscope, as the surgical area suffered a sudden high pressure expulsion of blood. After 10 minutes, 1200 ml blood had been lost and she lost consciousness due to acute shock. Her nasal cavity was packed with gauze and the bleeding temporarily stopped. A cerebral DSA (Digital Subtraction Angiography) was immediately performed. A pseudo-aneurysm of the left internal carotid artery (ICA) cavernous segment was revealed (Figure 3A arrow). When we tried to embolize it, a second bleeding occurred (Figure 3B), invading into cranial cavity, and she fell into deep coma. We immediately embolized the left ICA and performed vital resuscitation. Although supported by mechanical ventilation and circulation, she died one day later.

### Case 2

#### History and examination

A 49-year-old man had a one-year history of weakness in the lower limbs, walking instability, sporadic falling down, dizziness, nausea, and vomiting. His neurological examination revealed a tongue slant to right, a positive Romberg sign, and muscle strength grade IV in both lower extremities.An MRI scan revealed a large sub-tentorium lesion in the posterior fossa. The mass was sized 4.2 × 4.6 × 5.7 cm, affixed to the straight sinus and torcular, compressing forward. There was great displacement of the cerebellum, brain stem, and the fourth ventricle. The cerebella tonsillar had herniated into the foramen magnum. Supra-tentorial ventricular dilation was presented due to obstructive hydrocephalus. A T1-w MRI showed a slightly hypo-signal homogeneous tumor (Figure 1D), and T2-w and FLAIR(fluid-attenuated inversion recovery) scans revealed even hyper-signal. With gadolinium contrast, the lesion was obviously unevenly or partially enhanced, the enhancing was progressive from posterior to anterior, flame-like, with an irregular flocky twiggy edge, and the front part of the lesion was hardly enhanced (Figure 1E). The differential diagnosis included hemangioblastoma and meningioma.

#### Operation and postoperative course

A midline suboccipital craniotomy was performed. The tumor was found to be affixed to the torcular and the posterior part of the straight sinus, closely adhered with the dura. It was a purple color, tough, had a clear boundary, and an abundant blood supply. The tumor was removed piece by piece under a microscope. Most of the lesion was freed from the dura, except a minimal piece that could not be removed safely from the torcular, therefore a subtotal resection was made. Approximately 620 ml blood was lost altogether. The pathology features mimic those in case 1 (Figure 2C). The smooth muscle cells were positive for SMA (Figure 2D) and Vim, and the inner-vessel endothelial cells were positive for CD34, negative for epithelial membrane antigen (EMA), and negative for inhibin-a. The patient recovered without incident and an MRI scan showed a satisfying resection (Figure 3B). On a follow-up CT scan five months later and a further follow-up MRI scan one year later (Figure 1F), no recurrence was demonstrated. The patient’s legs resumed normal strength and recovered well, except for some walking instability.

### Case 3

#### History and examination

A 77-year-old man suffered from an intermittent headache for five months. His neurological physical examination was unremarkable. T1-w MRI scans revealed an iso-signal tumor arising from the diploic space of the left temporal skull vault, invading inward to temporal lobe, sized 1.6 × 3.1 × 3.9 cm. T2-w and FLAIR scans showed a hyper-signal lesion (Figure 1G). Obvious enhancement was seen after a gadolinium injection (Figure 1H). There was no enhancement of the dura or ‘dura tail’. The diploic angioma, meningioma, fibrosarcoma, and aneurysmal bone cyst were considered.

#### Operation and postoperative course

A craniotomy was performed. The cranial outer table was intact, the inner lamina and diploe were eroded with hyperostosis around. The tumor was a dark reddish hue with a rich blood supply, adhered to the dura. The whole tumor was removed. After autoclaving, the bone flap was returned and fixed in the original place. A histological analysis demonstrated typical elongated smooth muscle cells and dilated irregular vascular channels (Figure 2E). The smooth muscle cells were immunoreactive for SMA, actin, and Vim. The intra-vessel endothelial cells were positive for CD34 (Figure 2F). The patient recovered rapidly. At 12-month follow-up, MRI scans were performed, there was no tumor recurrence (Figure 1I).

## Discussion

ALM is a benign, vascular, smooth muscle tumor, commonly developing in the subcutaneous tissues of the lower extremities; an intracranial location is extremely rare. The first case was reported in 1994 by Lach *et al*. [7]. Since then, 12 cases have been subsequently described, and our current data is the largest series ever since the first report. In all the available cases (see Table 1), the male/female ratio is 12/3, not like the subcutaneous counterpart which has a female bias [1,8]. According to the 2002 World Health Organization classification of soft tissue tumors, ALM is recognized as an independent tumor entity. Its histological features are mainly composed of an abundant number of vascular channels separated by loosely organized smooth muscle bundles with a variable amount of collagen. SMA (smooth muscle actin) as a bio-marker of muscle cells, and CD34 for the endothelium of vessels, are essential for diagnosis. The histogenesis of these lesions is not clear.

**Table 1:** Literature review for the intracranial angioleiomyoma

**Author/Year**	**Age/Sex**	**Clinical symptoms**	**Site and size**	**Surgery**	**Follow-up**
Lach *et al*. [7]	47, M	Gait abnormality with a right-sided limp for 14 months.	In the leptomeninges of the right parietal lobe, No attachment to dura. 2 cm.	TR.	4 yrs. NR.
Ravikumar *et al.*[5]	12, F	Headache, seizures, left hemi-dystonia, and apraxia of eyelid closure.	Right head of caudate, large and cystic. The 2^nd^ lesion at left globus pallidus.	The right one: TR *en bloc*.	20 mths. NR. Left lesion quiescent
Karagama *et al*. [9]	47, F	Progressive hearing loss for 18 months.	Left auditory meatus. 1 cm.	TR.	1 year. NR.
Figueiredo *et al*. [3]	52, M	Horizontal diplopia and headache for 2 yrs. Facial numbness and impaired visual acuity for 6 mths.	Right cavernous sinus, 6.0 × 6.0 × 5.0 cm. Eroding the peripheral bone structure.	TR. Piecemeal resection, Severe bleeding	Not mentioned.
Colnat-Coulbois *et al*. [2]	50, M	Vertical diplopia for left trochlear nerve palsy.	Left cavernous sinus. About 3 cm.	TR, Venous bleeding	6 yrs. NR.
Gasco *et al.*[10]	43, M	Headache, blurred vision, dizziness, gait abnormalities.	Left cerebellar lesion attached to dura. 4.4 × 3.9 × 3.9 cm.	TR.	NR.
Xu *et al*. [6]	53, M	Headache for 3 months, visual deterioration.	Intra-sella, cystic, less than 1 cm.	TR.	NR.
Zhou *et al*. [13]	62, M	A sudden seizure.	In the middle cranial fossa, near cavernous sinus. 3.7 × 3.5 × 3.3 cm.	Not mentioned.	7 mths. NR.
Conner *et al*. [11]	42, M	8-year history of poorly localized headaches.	Infratentorial and located near the incisura, 0.8-1.0 cm.	TR *en bloc* with minimal bleeding	1 yr and 11 mths, NR.
Conner *et al*. [11]	36, M	8-year history of worsening daily headaches.	Falcine, posterior to the splenium, 2.5 cm.	Subtotal resection.	2 yrs and 2 mths, NR.
Shinde *et al*. [4]	60, M	Headache, seizures, and irritability for 2 mths.	Right putamen (2 cm). Left hippocampus, bilateral optic nerves and thickened meninges.	No surgery.	Died of recurrent seizures and septicemia.
Lescher *et al*. [12]	40, M	Focal epilepsy for 2 years.	In the falx cerebri, about 2 cm.	TR *en bloc*.	Not mentioned.
Current case 1	51, F	Visual deterioration for 2 months.	Intra-sella 3 cm.	TR with severe bleeding.	Died of delayed hemorrhage
Current case 2	49, M	Weakness of lower limbs for 1 year.	Sub-tentorium, affixed to torcular. 4.2 × 4.6 × 5.7 cm.	Subtotal R. severe bleeding	1 year, NR.
Current case 3	77, M	Headache for 5 months.	Left temporal diploic space, 1.6 × 3.1 × 3.9 cm.	TR.	1 year, NR.

F: female; M: male; TR: total resection; mths: months; NR: No recurrence; yrs: years. Age is presented in years.

The reported cases developed often in the dura matter, typically near large venous vessels like venous sinus or skull diploic structure, Our intra-sella case 1 involved the medial side of cavernous sinus and case 2 showed a large lesion in posterior cranial fossa arising from straight sinus and torcular. Figueiredo *et al.*[3] and Colnat-Coulbois *et al.*[2] reported para-cavernous lesions. Gasco *et al.*[10] reported a large ALM originated from the transverse sinus. A few cases reported small nodules on the dura [6,11,13], and a peculiar case in auditory meatus [9]. Some ALMs occur on cranial bone, with the majority were in the facial bones such as the mandible and maxilla [14]. Vijayasaradhi *et al.*[15] reported a lesion in the low frontal bone which eroded the outer table only, growing outward. In case 3, the inner table was eroded and the tumor invaded inward. Other than these ex-axis lesions, two exceptional cases should be mentioned which are primary multi-centric intra-parenchyma ALMs. Ravikumar *et al.*[5] reported a patient with two lesions, a large and cystic one in the right head of caudate and a solid one in the left globus pallidus. Shinde *et al.*[4] in 2012 described a primary multicenter ALM distributing in the right putamen (2 cm), left hippocampus, bilateral optic nerves, and thickened meninges. The clinical signs and symptoms are diverse, which depend on the anatomical location and the size of the lesion. Nonetheless, the common character of all these cases is that the tumor is benign, grows slowly, with no intracranial hemorrhage, no recurrence, or malignant transformation.

The imaging of ALM is confusing, the differential diagnosis of lesion with similar feature includes all extra-axis enhancing tumors such as meningiomas, pituitary tumor, angiopericytomas, cavernous hemangiomas, and schwannomas. By comparing the MRI scans of our presented cases, some common characters of ALM can be identified. The tumor shows a hyper-signal on T2-w and T2-FLAIR images and an iso-signal on T1-w images, with a flame-like partial enhancement after the injection of gadolinium. The ‘enhancement flame’ seemed to rise from the tumor base, while not reaching the far end of the tumor. Case 1 and case 2 are dramatically similar in this way. This phenomenon can also be seen in the image provided by Colnat-Coulbois *et al.*[2]. Their explanation is a ‘delayed progressive enhancement’, which means the tumor will be fully enhanced when enough time is given. The exact reason of this imaging is not clear now, in our opinion it may be due to its distinguishable blood vessel structure, which does not exist in other tumors. All in all it should be considered as a distinctive diagnostic clue. This phenomenon has not been formally presented and emphasized enough in the available literature.

Histological differential diagnosis of ALMs included other intracranial entities such as glioblastoma multiform (GBM), arteriovenous malformation (AVM), cavernous hemangioma, angiofibroma (AFM), and myopericytoma (MPC). As an infiltrating high-grade glioma, GBM is often a necrotic and cystic lesion, with high metabolism and high proliferation ability. It is usually GFAP-positive, has no diffused vessel structure, and mitotic figures can be seen. In our ALM cases, after meticulous searching, no mitoses was found, the Ki67-/MIB-index was very low, and GFAP was always negative. AVMs are described as abnormalities consisting of tangled masses of tortuous arteries, veins, and abnormal connecting channels. In the cavernous hemangioma we can also see extremely distant blood vessels or a blood-filled sinus. In ALMs, CD34 is positive in the vessel endothelium, SMA is positive in the smooth muscle bundles, and (80 to 90% of cases) desmin is also frequently immuno-positive [16]. Angiofibroma is most often found in the ‘juvenile nasopharyngeal angiofibroma’ (JNA) with intracranial extension, which usually develops in teenage boys, mostly in the nasopharyngeal region. JNA has an abundant vessel component but no smooth muscle (CD34 positive, SMA negative). Primary intracranial myopericytoma (MPC) is even rarer than ALM. The histological features of the two tumors may present similarly as both have myoid-appearing cells and a diffused vessel structure. However, MPC have oval-to-spindle shaped cells with a striking tendency for concentric perivascular growth [16], which can be distinguished immediately, and the neoplastic cells are usually negative for desmin antibodies.

Although ALM is benign and seemed to be easily curative by surgery in the literature, we have to say, for the abundance of vascular structure, surgery on ALM in certain locations is difficult and risky, especially when the tumor is rather large. In case 1, the operation was difficult and the delayed fatal complication was extremely rare. The reason of the formation of the pseudo-aneurysm was not clear, lessons should be drawn from the catastrophic outcome. The tumor might have eroded the wall of ICA, the risk of potential postoperative hemorrhage should be considered, and an earlier angiography could have avoided the fatal outcome. In case 2, as a large vascular mass in the posterior fossa, there was heavy blood loss in order to excise it piecewise. Figueiredo *et al.*[3] also reported severe blood loss in an excision of a 6 cm cavernous ALM. Now the preoperative polyvinyl alcohol (PVA) embolism has been commonly used in the patients with juvenile nasopharyngeal angiofibroma in many hospitals, the similar preoperative endovascular embolism may be a possible way to reduce the intraoperative bleeding of large lesions. When the intracranial ALMs are too dangerous to operate, radiotherapy becomes an alternative treatment option.

## Conclusions

Intracranial ALMs are extremely rare and are usually located on the ex-neuro axis, such as in our cases (the sella, posterior fossa, and the skull). MRI scans revealed a special feature of flame-like partial enhancement that may be helpful to distinguish ALM from pituitary tumors or meningiomas, and should result in the consideration of this tumor entity early in the diagnostic process. A definitive diagnosis depends on a histological analyses. The resection of ALM in certain locations is difficult and risky because of the rich blood supply.

## Consent

Written informed consent was obtained from the patient for publication of this case report and any accompanying images. A copy of the written consent is available for review by the editor-in-chief of this journal.

## Abbreviations

ALM: Angioleiomyoma; AVM: arteriovenous malformation; CT: Computed tomography; DSA: Digital subtractive angiography; FLAIR: fluid-attenuated inversion recovery; GBM: glioblastoma multiform; GFAP: glial fibrillary acidic protein; ICA: internal caratid artery; NF: neurofilament; H&E: Hematoxylin and eosin; JNA: juvenile nasopharyngeal angiofibroma; MPC: Primary intracranial myopericytoma; MRI: Magnetic resonance imaging; PVA: polyvinyl alcohol; SMA: Smooth muscle actin; Vim: vimentin.

## Competing interests

The authors declare that they have no competing interests.

## Authors’ contributions

LS reviewed the literature and drafted the manuscript. YZ and LS were clinically responsible for patient care. HW directed the operation and revised the manuscript. All authors read and approved the final manuscript.
